# Effect of Attenuated Light Through Translucent Zirconia on the Interfacial Adaptation and Polymerization of Resin Cements

**DOI:** 10.3290/j.jad.b4586857

**Published:** 2023-11-01

**Authors:** Seung-Hoon Han, Yasushi Shimada, Alireza Sadr, Tomoko Tabata, Hisaichi Nakagawa, Takaaki Sato, Ji-Eun Byun, Sung-Ho Park

**Affiliations:** a Associate Professor, Department of Conservative Dentistry, St. Vincent Hospital, College of Medicine, The Catholic University of Korea, Seoul, Republic of Korea. Idea, hypothesis, experimental design, performed the experiments in partial fulfillment of requirements for a degree, wrote the manuscript.; b Professor, Department of Cariology and Operative Dentistry, Graduate School of Medical and Dental Sciences, Tokyo Medical and Dental University (TMDU), Tokyo, Japan. Performed the experiments in partial fulfillment of requirements for a degree, proofread the manuscript, contributed substantially to discussion.; c Associate Professor, Department of Restorative Dentistry, School of Dentistry, University of Washington, Seattle, WA, USA. Proofread the manuscript, consulted on and performed statistical evaluation, contributed substantially to discussion.; d Assistant Professor, Department of Cariology and Operative Dentistry, Graduate School of Medical and Dental Sciences, Tokyo Medical and Dental University (TMDU), Tokyo, Japan. Performed the experiments in partial fulfillment of requirements for a degree, performed a certain test.; e Assistant Professor, Department of Oral Biomedical Engineering, Graduate School of Medical and Dental Sciences, Tokyo Medical and Dental University (TMDU), Tokyo, Japan. Performed the experiments in partial fulfillment of requirements for a degree, performed a certain test.; f Lecturer, Department of Oral Biomedical Engineering, Graduate School of Medical and Dental Sciences, Tokyo Medical and Dental University (TMDU), Tokyo, Japan. Performed the experiments in partial fulfillment of requirements for a degree, performed a certain test.; g PhD Student, Department of Conservative Dentistry, College of Dentistry, Yonsei University, Seoul, Korea. Performed the experiments in partial fulfillment of requirements for a degree, performed a certain test.; h Professor, Department of Conservative Dentistry, Oral Science Research Center, College of Dentistry, Yonsei University, Seoul, Korea. Contributed substantially to discussion.

**Keywords:** interfacial adaptation, translucent zirconia, resin cement, polymerization shrinkage strain, exposure reciprocity, radiant exposure window

## Abstract

**Purpose:**

The first objective was to determine if dual-curing of resin cement with reduced light could affect interfacial adaptations of zirconia restoration. The second objective was to examine whether cement type and pretreatment method of universal adhesive affected interfacial adaptation. The final objective was to compare the polymerization degree of cement under different reduced-light conditions.

**Materials and Methods:**

Inlay cavities were prepared on extracted third molars. Translucent zirconia restorations were milled using Katana UTML (Kuraray Noritake) in three groups with restoration thicknesses of 1, 2, and 3 mm, respectively. Each group had three subgroups using different cementation methods. For subgroup 1, restorations were cemented with self-adhesive cement. For subgroup 2, universal adhesive was applied and light cured. After the restoration was seated with conventional resin cement, light curing was performed. For subgroup 3, after adhesive was applied, the restoration was seated with conventional resin cement. Light curing was performed for the adhesive and cement simultaneously. After thermocycling, interfacial adaptation at the restoration-tooth interface was investigated using swept-source optical coherence tomography imaging. Finally, polymerization shrinkage of the cement was measured using a linometer and compared under the conditions of different zirconia thicknesses and light-curing durations.

**Results:**

Interfacial adaptation varied signficantly depending on the zirconia thickness, pretreatment, polymerization mode and cements used (p < 0.05). The effects of the adhesive and polymerization shrinkage differed signficantly, depending on the reduced light under the zirconia (p < 0.05).

**Conclusion:**

Lower curing-light irradiance may lead to inferior adaptation and lower polymerization of the cement. Polymerization of resin cement can differ depending on the light irradiance and exposure duration.

Among dental restorative materials, zirconia is one of the least translucent.^65^ When light curing is applied over the zirconia restoration, the resin cement receives reduced light irradiance, which may lead to insufficient polymerization. When conventional resin cement is used, dentin adhesive should be applied prior to cement placement. It has been reported that dentin adhesive should be polymerized immediately by light curing.^39^ When light curing is difficult, two bottles of adhesive – including base and catalyst – have been provided for the adhesive to be self-cured. Recently, some manufacturers have suggested that their single-bottle adhesive can self-cure both the adhesive and cement. In these systems, accelerators are included in the adhesive to self-cure the cement paste, and activators are present in the cement paste to self-cure the adhesives.

Dentin adhesive for resin cement can be light cured by two different methods: a pre-cure or a co-cure method. In the pre-cure method, the adhesive is light cured before cement placement. Studies showed that pre-curing of the adhesive improved marginal adaptation and increased the bond strength compared to that of co-curing.^21,45^ In the co-cure method, the adhesive is not light cured prior to placement of the cement, but instead, after the adhesive is applied and the cement is placed with the restoration, the adhesive and cement are light cured at the same time. When a restoration is cemented by the pre-cure method, precautions should be taken. If a small amount of adhesive pools in the line angles of the prepared cavity, and is cured as is, the pre-cured adhesive may interfere with accurate restoration seating. Thick pre-cured adhesive layers are more prone to this problem. The thickness of the adhesive layer depends on the composition of the adhesive.^7,11^ One study demonstrated that adhesive layer thickness decreased linearly with increasing solvent (acetone) content.^10^ To avoid inaccurate fitting, the co-cure method may be preferable in clinical situations. Therefore, for good bonding without interference, the optimal conditions for the co-cure method need to be identified.

Under zirconia restorations, the irradiance of the curing light can be reduced, depending on the type and thickness of the material.^62^ Longer irradiation time is required to compensate for reduced irradiance, and to provide sufficient radiant energy.^24,32^ It remains unclear whether a high degree of polymerization can be achieved when light with a lower irradiance is applied for a sufficient period. Previous research has presented divergent opinions on this topic. First, the total energy principle (or exposure reciprocity) states that similar radiant exposure leads to a similar polymerization and mechanical properties.^15,25^ However, other studies have shown that irradiance and curing time have separate effects on the polymerization process. This means that even the same radiant exposure can result in different material properties depending on the light irradiance and curing time.^20,43,48^ Thus, it needs to be determined whether extended light-curing time can compensate for reduced irradiance in the polymerization of resin cements.

To verify exposure reciprocity, the degrees of conversion can be compared.^25,55^ It can be defined as the extent to which monomers react to form polymers, or as the proportion of C=C double bonds converted into C-C single bonds. The degree of conversion can be measured using direct and indirect methods, such as Fourier-transform infrared spectroscopy (FTIR), Raman spectroscopy, differential scanning calorimetry (DSC), microhardness testing, or polymerization shrinkage measurement.^14,18,48,54^

Polymerization shrinkage strain is an inherent feature of resin composites.^2,9^ Polymerization shrinkage creates detrimental stress at the restoration-tooth interface, which may lead to adhesion failure. Polymerization shrinkage strain increases in proportion with the degree of conversion.^4,52^ When shrinkage strain is measured in real time, the polymerization reaction rate or speed can also be calculated. However, the shrinkage strain of a resin cannot be directly compared to that of another resin, because the correlation between the degree of conversion and polymerization shrinkage is material dependent.^13^ One study reported that if some carbon double bonds form small segmental rings, shrinkage strain will show different correlations.^60^ For this reason, polymerization shrinkage of one resin cannot be compared with that of another, but can be compared under different conditions using the same material.

Optical coherence tomography (OCT) is based on low-coherence interferometry, and presents non-destructive images.^27,44^ When a laser beam is projected over a tooth or restoration, the backscattered light is transformed into a signal intensity.^8^ When the light of OCT passes the interface between two media with different refractive indices, a portion of light is reflected. The reflected light can make the OCT indicate a different signal intensity. By means of OCT, the restoration-tooth interface can be examined without damaging a specimen. Swept-source optical coherence tomography (SS-OCT) is one type of OCT that has higher speed and better image resolution.^56^

The first aim of the present study was to evaluate whether there would be a difference in interfacial adaptation when resin cements were polymerized by attenuated light through tranlucent zirconia, or polymerized by self-curing. The second aim was to compare a self-adhesive resin cement (SAC) and a conventional resin cement after two different adhesive pretreatments: pre-cure and co-cure. The final aim was to compare the polymerization shrinkage strain (PSS) given attenuated light under different thicknesses of zirconia and under different durations of light exposure.

The null hypotheses are as follows:

1. There is no difference in interfacial adaptation of translucent zirconia restorations when the resin cements are dual cured with light attenuated through different zirconia thicknesses or self-cured.
2. The interfacial adaptation of translucent zirconia restoration does not differ with SAC or conventional resin cement using the pre-cure and co-cure method.
3. There is no difference in polymerization shrinkage strain (PSS) when the cement was dual cured by different combinations of reduced irradiance and exposure duration, as long as the same radiant exposure is applied.

## Materials and Methods

### Measurement of Irradiance under Normal and Attenuated Light Conditions

The first step gauges the irradiance (radiant exitance) of a light-curing unit (LCU) under normal light conditions and under attenuated light conditions. The effect of reduced (attenuated) light was investigated not by light reduced at the light source, but by light reduced by penetrating through different thickness of zirconia. The irradiance was measured using an LCU (Elipar S10, 3M Oral Care; St Paul, MN, USA) under seven conditions: one normal condition and six reduced-light conditions through different thicknesses of zirconia. A spectrometer (Flame-T, Ocean Insight; Largo, FL, USA) and an integrating sphere (Labsphere; North Sutton, NH, USA) were used. After the radiant flux between 360 and 540 nm was measured, the radiant exitance was calculated to be 1176 mW/cm^2^ for the normal condition. The reduced irradiances were measured through 10 x 10 mm zirconia blocks (Katana UTML, A2 shade, Kuraray Noritake; Tokyo, Japan) with thicknesses of 0.5, 1, 2, 3, 4, and 5 mm. After positioning the zirconia block between the integrating sphere aperture and the light-curing tip, radiant flux was measured and the irradiance was calculated. Each measurement was repeated ten times and the mean was calculated.

### Tooth Preparation

**Fig 1:**
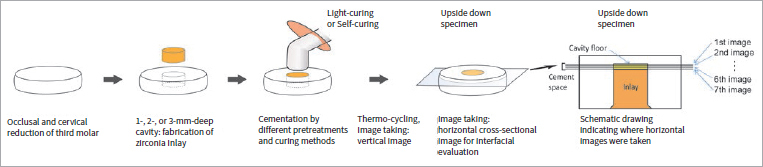
Experimental procedure for the evaluation of restoration–tooth interfacial adaptation.

The setup employed in this study is schematically illustrated in Fig 1. The Institutional Review Board granted use of the teeth under approval number of VC20TISI0002. Extracted human third molars were stored in a 0.5% chloramine solution at 4°C. Seventy-two teeth were chosen that were free of caries. The occlusal surface of the tooth was flattened with a trimmer, 320-grit, and 600-grit SiC papers. Cylindrical class-I cavities were prepared with a diamond bur (Mani; Shioya, Japan) and a stone point (Dura-Green Dia TC4, Shofu; Kyoto, Japan). The dimensions were 3 (±0.2) mm in diameter and 1, 2, or 3 (±0.1, each) mm in depth depending on the group. The tooth specimen was cut 1 mm below the cementoenamel junction. Then, the cervical base of each tooth was also flattened until the remaining dentin thickness of the cavity base was 0.4 mm.

### Zirconia Inlay Fabrication

The preparation was scanned optically and an inlay was fabricated with a 100-μm cement space (Medit Identica hybrid scanner and milling software Excad v 2.0.0.3, Medit; Seoul, Korea). Highly translucent zirconia (Katana UTML, A2 shade, Kuraray Noritake) was used for fabrication of the inlay. Zirconia inlays were milled at the center position of the zirconia disk (transition layer) with a Roland Inlab milling machine (DWX-51D, Roland DG; Hamamatsu, Japan). The milled inlays were sintered in a furnace at a rate of 15°C/min to a temperature of 900°C, followed by a rate of 5°C/min to a temperature of 1250°C, and finally at a 3°C/min rate to a temperature of 1500°C. After sintering, the inlays were finished to be fitted into the prepared cavity.

### Restorative Procedure for Dual-Curing in Reduced Light (DC) and Self-cure (SC) Groups

This study evaluated the effect of their different bonding protocols on restoration adaption. The cements were polymerized under the conditions of 12 subgroups, which were composed of nine DC subgroups and three SC subgroups (n = 6 for each subgroup). This experiment evaluated the self-adhesive resin cement RelyX U200 (3M Oral Care) and the conventional resin cement RelyX Ultimate (3M Oral Care) after a different pretreatment with a universal adhesive (Single Bond Universal, 3M Oral Care). For the restoration, the internal surface of the restoration was air-blasted with 50-µm aluminum oxide particles (Hi Aluminas, Basic material; Renfert, Germany) under 30 psi of pressure for 20 s. The restoration was ultrasonically cleaned in water and dried. Then, a zirconia primer (Z-primer, Bisco; Schaumburg, IL, USA) was applied and dried.

### Dual-cure (DC) Groups Depending on Cavity Depth, Pretreatment, and Cement

For dual curing of the reduced light groups, the teeth were divided into three groups (I, II, and III) depending on the cavity depth. For Group I, the tooth was prepared with a 1-mm depth and a 3-mm diameter (n = 18). For Group II, the tooth was prepared with a 2-mm depth and a 3-mm diameter (n = 18). For Group III, the tooth was prepared with a 3-mm depth and a 3-mm diameter (n = 18). For each group, the teeth and milled restorations were randomly assigned to three subgroups (n = 6 per subgroup). The three subgroups (1, 2, and 3) differed depending on the type of resin cement and pretreatment.

For subgroup 1, a fabricated restoration was cemented using self-adhesive resin cement (SAC) with no pretreatment. The prepared surface of the tooth cavity was swabbed with wet cotton and gently air blasted.^47^ After RelyX U200 was mixed and loaded into the cavity, the inlay was positioned. Light was applied using the same LCU for 20 s. For subgroup 2, the restoration was cemented by the pre-cure method using adhesive and a conventional resin cement. A universal dentin adhesive (Single Bond Universal, 3M Oral Care) was applied on the prepared tooth cavity for 20 s. The adhesive was mildly air blown. Extreme care was taken that the adhesive did not pool at the line angles of the prepared cavity. Then, the adhesive was light cured for 10 s. The conventional resin cement (RelyX Ultimate, 3M Oral Care) was mixed and loaded. After the seating procedure, the restoration was light cured. For subgroup 3, the restoration was cemented by the co-cure method using the same adhesive and cement as those of subgroup 2. The adhesive was applied into the cavity. The adhesive was not light cured before cement placement. RelyX Ultimate was mixed and loaded. After the placement of the restoration, light curing was performed. All specimens were kept in a dark room at 100% humidity and 23±1°C.

### Self-cure (SC) Groups

For the self-cure groups, all cements were polymerized by the self-curing method. Subgroups differ depending on the cement and pretreatment method, as the case in dual-cure groups.

For self-cure group 1, a 3-mm-thick zirconia restoration was cemented with the SAC and allowed to self-cure (SC1, n = 6). The RelyX U200 (RU2) cement was mixed and loaded into the prepared cavity. The restoration was seated and the cement self-cured. For self-cure group 2 (SC2, n = 6), the restoration was cemented by the pre-cure method. Single Bond Universal (SBU) adhesive was applied and pre-cured using light. The RelyX Ultimate (RXU) cement was mixed and loaded. After placing the restoration, the cement was self-cured. For self-cure group 3 (SC3, n = 6), the restoration was cemented by the co-cure method. SBU adhesive was applied and dried, but not light cured. After RXU cement was loaded and the restoration placed, the cement was self-cured. The specimens were kept in the dark at 100% humidity and room temperature (23±1°C).

Note that subgroup 1 specimens of each group were created in a previous study^29^ conducted by our research group at this research institution using the same experimental protocol. The results of subgroup 1 were adopted from our prior study and applied here.

### Thermocycling Procedure

Each tooth specimen was kept in water at room temperature (23±1°C) after 24 h of finishing cementation. Then, each specimen was thermocycled 10,000 times (Thermal cycling tester RB 508, R&B; Daejeon, Korea), which was estimated to simulate clinical function of about one year.^22^ The teeth were immersed in water baths of 5°C and 55°C, with a transfer time of 5 s and a dwell time of 30 s. They were kept in water at room temperature (23±1°C) after thermocycling.

### Image Acquisition by SS-OCT and Image Analysis

For image acquisition, a swept-source optical coherence tomography (SS-OCT, IVS-2000, Santec; Komaki, Japan) was used. The axial resolution was 7 µm in hard tissues and resin composites, assuming a refractive index of 1.5. The specimen was placed upside down on the OCT device platform, and the scanning probe was placed above its base. In vertical cross-section, the SS-OCT image was produced at the center of the restoration, parallel to the bucco-lingual plane of the tooth. This image was used as the reference. In the horizontal cross-section, the first SS-OCT image was obtained parallel to the cavity floor at 5 µm below the cavity base. The second image was captured 15 µm below the first. For each specimen, seven images were collected at 15 µm intervals in a 100 µm cement space (Fig 1).

**Fig 2:**
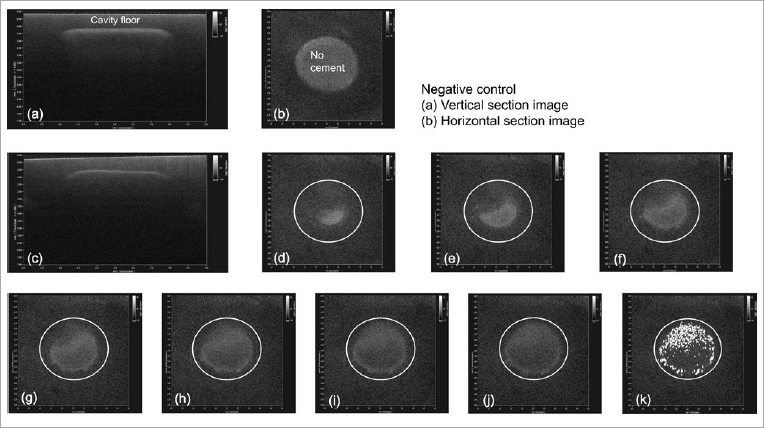
Representative SS-OCT images for interfacial adaptation evaluation of upside down specimens. (a) Negative control image (without cement): vertical cross-section. (b) Negative control image: horizontal cross-section. (c) Lateral cross-sectional image of a specimen in Group III. (d) The first horizontal cross-sectional image in the cement space after cementation with RXU. The white circle represents the border of a prepared cavity. The first image was taken parallel to the cavity floor at a level of 5 µm down from the cavity base. (e) The second image in the cement space. The second image was taken parallel at a level of 15 µm down from the first image. (f), (g), (h), (i) and (j) The 3rd, 4th, 5th, 6th and 7th images, respectively, were taken parallel at a level of 15 µm down from the previous image. (k) The same image as in (j) processed by GapAnalyzer. The white dots on the image (k) are brighter pixels which have a higher signal intensity than the threshold to indicate a microgap. On image (k), the interfacial adaptation (HB%) was calculated. The area of white dots was measured and then divided by the circle area. On the image of (k), the HB% was measured to be 24.7%. Procedural modifications were made from a previous study^28^ because the specimen was placed upside down.

Interfacial adaptation of the restoration and tooth were evaluated using image analysis software (ImageJ v. 1.49, National Institutes of Health; Bethesda, MD, USA). If a gap existed at the restoration–tooth interface, a portion of the light could be reflected from the interface. Consequently, the SS-OCT would show it as a bright area or spot on the image (Fig 2). The HB% (high brightness) parameter was created to represent the percentage of microgap at the cement space. The brighter pixels with higher signal intensity above the threshold were measured on the image using a plug-in program, GapAnalyzer, coded by one of our co-authors.^8,64^ The mean HB% was calculated for each specimen using seven horizontal images, with a higher HB% indicating inferior interfacial adaptation.

### Polymerization Shrinkage Strain (PSS) of the Resin Cements

#### 1. Calculation of radiant exposure and equivalent light duration

To verify the exposure reciprocity rule, the radiant exposure of each group was calculated first: the product of irradiance (W/cm^2^) under each thickness of zirconia and light exposure time (20 s) was calculated (Table 1). Next, when 3-mm-thick zirconia was used to reduce light, the required duration of light exposure was calculated to have the same radiant exposure for each group (Table 1, last row). To calculate the required duration of light, which was termed “equivalent light duration” in this study, the radiant exposure (J/cm^2^) of each group was divided by the irradiance under 3-mm-thick zirconia (W/cm^2^).

**Table 1:** Irradiance (radiant exitance) measured through each thickness of zirconia (Zr) block, radiant exposure and equivalent light duration


Irradiance measured through different thickness of zirconia (Zr) block	Zr block thickness	5 mm	4 mm	3 mm	2 mm	1 mm	0.5 mm	No block
	Irradiance (mW/cm^2^)	35.2	56.1	98.6	174.1	320.2	489.7	1176
Radiant exposure (light exposure duration of 20 s)	Radiant exposure (J/cm^2^)	0.7	1.1	2.0	3.5	6.4	9.8	23.5
Calculated duration of reduced light exposure under 3 mm Zr	Equivalent light duration (s)	7.1	11.2	20	35.3	64.9	99.3	239

The result of irradiance is the mean of ten measurements. Radiant exposure is the product of irradiance (W/cm^2^) and the light exposure duration (s). Equivalent light duration is the required exposure time to achieve the same radiant exposure for each group (for each column). To calculate it, the radiant exposure (J/cm^2^) of each group is divided by the light irradiance under 3 mm thick zirconia (W/cm^2^).

#### 2. Polymerization shrinkage by direct light exposure (positive control, PSS-T0D20)

Polymerization shrinkage strain (PSS) of the two resin cements, RU2 and RXU, were measured using a custom-made linometer (R&B), based on that presented by de Gee.^13^ First, the glass slide and disk were coated with high-vacuum grease (DowCorning; Midland, MI, USA). Resin cement, 5 mm in diameter and 1 mm in height (0.05 g), was mixed, then placed onto a metal disk, and covered with a slide glass. Measurement was initiated at the time of light curing, which was 90 s after the start of mixing cement. The LCU (Elipar S10) was positioned over the slide glass for direct light curing. Light was applied for 20 s. The metal disk moved upward as the resin cement was polymerized. The amount of linear displacement (ΔL) was measured by an eddy current sensor at room temperature (23±1°C) for a period of 15 min. Then, the volumetric shrinkage of polymerization (PSS) was calculated using the following equation.^13^

First, Lin% (linear shrinkage %) was calculated by:


$$\mathrm{Lin}\%=\frac{\Delta L}{L+\Delta L}\times 100$$

in which ΔL is the linear displacement of the disk and the L is the specimen thickness after polymerization.

Polymerization shrinkage strain (PSS) is given by:


$${\text{ PSS = 3Lin\% - 0.03(Lin\%) }}^{2}+0.0001(\mathrm{Lin}\%{)}^{3}$$

This measurement was repeated ten times and averaged (n = 10).

#### 3. Polymerization shrinkage strain (PSS) under reduced light penetration through different zirconia thicknesses

The PSSs of the two cements were measured under different levels of reduced light penetrating through a zirconia block. Resin cement was placed onto the disk with the same dimensions as described above. PSS-T5D20 is the PSS measured under reduced light through 5-mm-thick zirconia (T5) for 20-s light duration (D20). For PSS-T5D20, a Katana UTML sintered block (width x length x height: 10 x 10 x 5 mm) was placed on the slide. The LCU was positioned above the block, and the light was applied for 20 s. PSS was measured and calculated in the same way as the section above. For PSS-T4D20, PSS-T3D20, PSS-T2D20, PSS-T1D20, and PSS-T0.5D20, PSSs were measured under zirconia blocks of 4-mm, 3-mm, 2-mm, 1-mm, and 0.5-mm thickness for 20 s of irradiation, respectively. Measurements were taken ten times and averaged (n = 10).

#### 4. Polymerization shrinkage strain (PSS) under one reduced irradiance for different light-exposure durations

The PSS of the two cements were measured under one reduced irradiance for different light-exposure durations. Resin cement was mixed and placed as described above. A 3-mm-thick block (width x length x height: 10 x 10 x 3 mm) was positioned onto the slide glass. PSS was measured and calculated for different light durations. Duration of light exposure (curing time) was determined by calculating the equivalent light duration (the last row in Table 1). For PSS-T3D7, the light curing was performed over the 3-mm-thick block for 7 s. PSS-T3D11, PSS-T3D20, PSS-T3D35, PSS-T3D65 and PSS-T3D99 were measured with the reduced light through 3-mm-thick zirconia for irradiance durations of 11 s, 20 s, 35 s, 65 s, and 99 s. Measurements were taken ten times and averaged (n = 10).

#### 5. Polymerization shrinkage with self-curing (PSS-SC)

The PSS of the two cements were measured when self-cured. After the mixed cement was placed, the glass slide was covered to ensure darkness for self-curing. Light curing was not performed. Measurement was initiated at 90 s after the start of mixing the cement. Measurements were taken for 15 min and repeated ten times (n = 10).

### Statistical Analysis

Because the measurements were distributed normally, parametric statistical tests were used (Shapiro-Wilk test, p > 0.05). Levene’s test was used to test for homogeneity of variance (p > 0.05). The HB% of interfacial adaptation was evaluated using two-way ANOVA to check the effect of irradiance, cementation method, and their interaction. To compare the effects of reduced irradiance, one-way ANOVA and Scheffe’s test were conducted (Table 2, each row). To compare the effects of cement and cementation method, one-way ANOVA and Scheffe’s test were also performed (Table 2, each column).

**Table 2:** Interfacial adaptation (HB%) at the restoration–tooth interface after thermocycling of the specimens

	Group I (1 mm DC)	Group II (2 mm DC)	Group III (3 mm DC)	Self-cure
RU2 with no pretreatment*	19.7 (3.8)^d,A^	22.8 (4.1)^c,A^	27.0 (4.1)^b,A^	31.9 (4.2)^a,B^
RXU with pre-cure method	15.8 (3.8)^c,B^	17.3 (3.7)^c,B^	22.0 (3.8)^b,C^	28.5 (4.0)^a,C^
RXU with co-cure method	16.3 (3.8)^c,B^	17.9 (3.5)^c,B^	24.2 (3.8)^b,B^	34.2 (4.1)^a,A^

Numbers in parentheses are standard deviations. DC: dual-cure. Higher HB% indicates inferior interfacial adaptation. Values followed by the same lowercase letters in each row are not significantly different (one-way ANOVA and Scheffe’s test, p > 0.05). Values followed by the same capital letters in each column are not significantly different (one-way ANOVA and Scheffe’s test, p > 0.05). Subgroup 1 results were adopted from the authors’ prior study^29^ conducted at this research institution using the same experimental protocol.

To compare the PSS of different radiant exposure, one-way ANOVA and Scheffe’s test were conducted (Table 3, each row of PSS). To verify the exposure reciprocity rule, PSS under different reduced-light levels was compared to that of different irradiation durations for each cement (Table 3, comparison between the first and second row results of PSS and between the third and fourth row results of PSS). For statistical comparison, independent t-tests were performed. All the statistical analyses was conducted using SPSS software (IBM SPSS Statistics v22, IBM; Armonk, NY, USA) with the significance level set at α=0.05.

**Table 3:** Polymerization shrinkage strain (PSS) percentage under the different reduced light and exposure durations

Radiant exposure for each group (for each column, J/cm^2^)			0.7	1.1	2.0	3.5	6.4	9.8	23.5
RU2	Zr thickness, duration	No light (SC only)	5 mm, 20 s	4 mm, 20 s	3 mm, 20 s	2 mm, 20 s	1 mm, 20 s	0.5 mm, 20 s	0 mm, 20 s
	PSS	2.08 (0.10)^f^	2.15 (0.12)^f,B^	2.70 (0.13)^e,A^	3.17 (0.12)^d^	3.54 (0.14)^c,A^	4.12 (0.14)^b,A^	4.36 (0.12)^a,A^	4.42 (0.13)^a^
	Zr thickness, duration	No light (SC only)	3 mm, 07 s	3 mm, 11 s	3 mm, 20 s	3 mm, 35 s	3 mm, 65 s	3 mm, 99 s	
	PSS	2.08 (0.10)^f^	2.33 (0.11)^e,A^	2.60 (0.12)^d,A^	3.17 (0.12)^c^	3.49 (0.13)^b,A^	4.05 (0.15)^a,A^	4.17 (0.13)^a,B^	
RXU	Zr thickness, duration	No light (SC only)	5 mm, 20 s	4 mm, 20 s	3 mm, 20 s	2 mm, 20 s	1 mm, 20 s	0.5 mm, 20 s	0 mm, 20 s
	PSS	1.99 (0.11)^f^	2.12 (0.12)^f,B^	2.82 (0.12)^e,A^	3.29 (0.13)^d^	3.82 (0.14)^c,A^	4.18 (0.13)^b,A^	4.43 (0.15)^a,A^	4.59 (0.12)^a^
	Zr thickness, duration	No light (SC only)	3 mm, 07 s	3 mm, 11 s	3 mm, 20 s	3 mm, 35 s	3 mm, 65 s	3 mm, 99 s	
	PSS	1.99 (0.11)^f^	2.29 (0.11)^e,A^	2.65 (0.11)^d,B^	3.29 (0.13)^c^	3.77 (0.12)^b,A^	4.14 (0.15)^a,A^	4.21 (0.16)^a,B^	

For each column, the PSS values were measured by light application of a fixed radiant exposure. Numbers in parentheses are standard deviations. To compare the PSS by radiant exposure, one-way ANOVA and Scheffe’s test were performed. Values in each row marked by the same lowercase letters are not significantly different (p > 0.05). To compare the PSS with reduced irradiance to that of different exposure durations, independent t-test was conducted for RU2 and RXU groups (comparison between the first and second row results of PSS and between the third and fourth row results of PSS). Values in each column marked by the same capital letters are not significantly different (p > 0.05). No light (SC only) indicates PSS measured under the self-curing condition.

## Results

Table 1 (first row) shows irradiance measured through each thickness of the zirconia block. The radiant exposure is presented in the second row considering 20 s of light application. Equivalent light duration is calculated and presented in the last row to have the same radiant exposure in each group (for each column).

**Fig 3:**
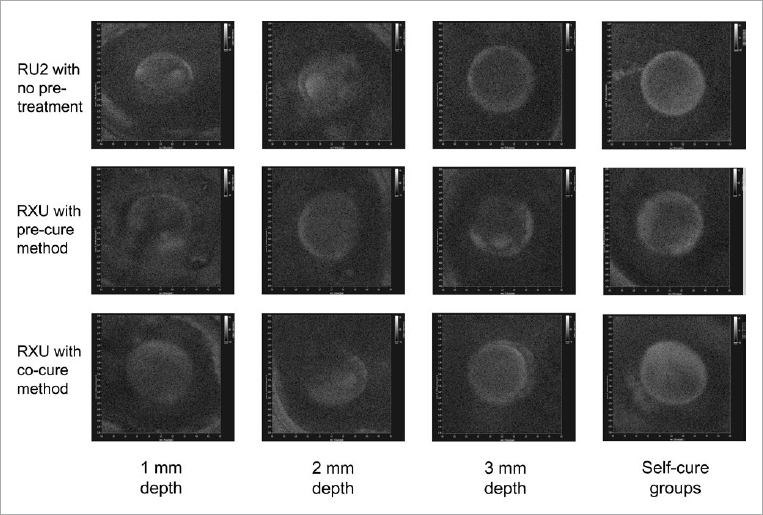
Representative cross-sectional images at the restoration–tooth interface. All the images were taken after thermocycling of the specimens. The first column shows the representative images of Group I (1 mm depth) including subgroups 1, 2, and 3. For subgroup 1, RU2 cement was used with no pretreatment. For subgroups 2 and 3, RXU cement was used with the pre-cure and co-cure methods. The second, third, and fourth column show the images of Group II (2 mm), Group III (3 mm), and self-cure (SC) including the same subgroups.

The interfacial adaptations (HB%) of the zirconia restorations are summarized in Table 2, and horizontal-sectional images are presented in Fig 3. The interfacial adaptation (HB%) is the measurement of microgaps, bubbles, or voids at the restoration–tooth interface after thermocycling of the specimens. For the HB% result, two-way ANOVA showed that the effects of the irradiance, cementation method and their interaction were all significant (p < 0.001, p < 0.001, and p < 0.05, respectively). After one-way ANOVA and Scheffe’s test, different irradiance (restoration thickness) led to differences in interfacial adaptation. Generally, an increased thickness of the restoration affected the interfacial adaptation negatively (Table 2, each row, p < 0.05). However, there were no differences between Group I and Group II with regard to pre- or co-cure with RXU (Table 2, p > 0.05). The results also showed that the activation mode (dual-cure or self-cure) affected the interfacial adaptation, with the self-cure groups showing a larger gap (Table 2, each row, p < 0.05). Regardless of the zirconia thickness, interfacial adaptation of the dual-cure groups was better than that of the self-cure groups.

The cementation method, which included cement type and universal adhesive pretreatment, led to different interfacial adaptations for each restoration thickness (Table 2, comparison in each column, p < 0.05). For the dual-cure groups, interfacial adaptation by conventional resin cement with universal adhesive was superior to that of SAC (comparisons between subgroup 1 and subgroup 2 or subgroup 3). The interfacial adaptation using the pre-cure method was similar to that of the co-cure method in Groups I and II; however, the interfacial adaptation of Group III pre-cure was different from that of Group III co-cure (Table 2, the third column). Self-cure groups showed different interfacial adaptation depending on the cement type and the pretreatment method (Table 2, the last column, p < 0.05).

**Fig 4:**
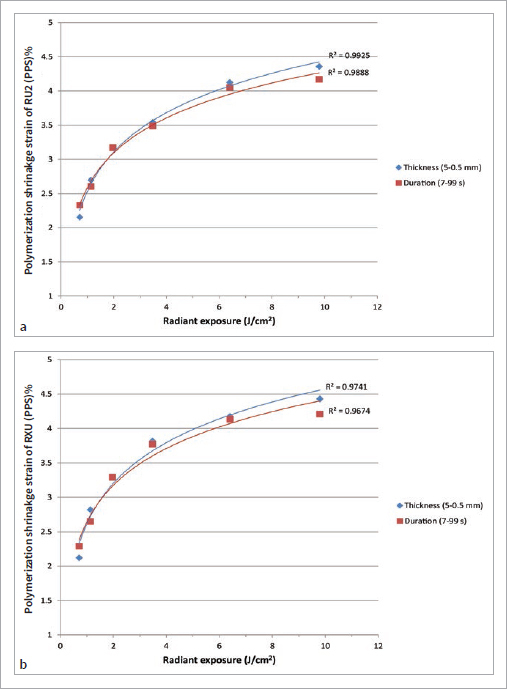
(a) represents the polymerization shrinkage strain (PSS) percentage as a function of the radiant exposure measured with self-adhesive resin cement (RU2). Each “thickness” dot (5–0.5 mm) indicates the mean PSS measured by each 20 s light duration and different levels of reduced light using various zirconia thicknesses. Each “duration” dot (7–99 s) indicates the mean PSS measured by one reduced light under 3 mm zirconia and different equivalent light duration. Each line of best fit (regression line) is presented in a logarithmic regression analysis. (b) Represents the PSS as a function of the radiant exposure measured with the conventional resin cement (RXU). Thickness, duration, and lines indicate the same as in (a).

Table 3 shows PSS measured given various combinations of different levels of reduced irradiance and light durations. For each column, the PSS values measured by light application of the same radiant exposure are presented. Increased radiant exposure led to higher PSS (Table 3, each row). The result showed that exposure reciprocity rule was valid in the mid-range of the radiant exposure (Table 3, comparison between the first and second row results of PSS and between the third and fourth row results of PSS). Figure 4 (a) and (b) show the relationship between the radiant exposure and the polymerization shrinkage strain (PSS). The lines of best fit (regression line) are presented in a logarithmic regression analysis.

## Discussion

The first null hypothesis was rejected, because interfacial adaptation under various zirconia thicknesses differed. The second null hypothesis was also rejected, because cement and pretreatment method affected the interfacial adaptation. The final hypothesis was rejected because the polymerization shrinkage strain (PSS), which was measured under the combinations of different levels of reduced light and exposure duration, differed in the lowest and highest radiant exposure groups.

Interfacial adaptation was worse in deeper cavities despite the lower polymerization shrinkage of resin cement. A possible reason is that lower light energy was delivered to resin cement (Tables 1 and 2). Low polymerization shrinkage helps reduce the polymerization contraction stress applied to the bonded interface. However, low polymerization of resin results in high solubility, residual monomer elution, and low physical properties, which can compromise the longevity of the restoration.^3,35^ For durable bonding, the polymerization of resin should be adequate; however, polymerization contraction stress should be reduced.^19^ In previous studies, the physical properties of specimens polymerized by self-curing were compared with those produced by dual-curing using reduced light.^1,33^ It was reported that the inferior results with self-curing can be due to a small number of free radicals and the low cross-linking density of the polymerized material.^5,37^ Self-curing results in lower cross-linking with fewer chain-growth centers and a more linear polymer structure, while light curing can produce more cross-linking.^6,41,59^ Other studies have shown that mechanical properties are highly influenced by the cross-linking density.^38,42,57^

The interfacial adaptation of subgroup 1 (RU2) differed from that of subgroups 2 and 3, which were cemented by conventional resin cement (RXU) and a universal adhesive (Table 2). Conventional resin cement is placed after application of the adhesive. Depending on the restoration thickness and polymerization mode, the pre-cure and co-cure methods produced different results. The interfacial adaptation of co-cure was inferior to that of pre-cure in Group III. The attenuated light seems to be an important factor in this situation. Under a thick restoration, the pre-cure method is recommended, since it can provide the adhesive layer with minimally attenuated light, which would result in better polymerization of the adhesive layer.

Simultaneous self-curing of dentin adhesive and resin cement showed the most inferior interfacial adaptation (Table 2, last column). Recently, some resin cements have been introduced with a one-bottle dentin adhesive, which is proposed to be self-curing in a single application. In this experiment, incomplete polymerization of the adhesive and/or cement may be a contributing factor for the poor adaptation in the self-cure groups.^40,63^ Another reason may be possible acid-base reactions between the remaining acidic composition in the adhesive layer and the basic component in the cement paste.^61^ Finally, poor adaptation may be due to the conditions under which the specimens were kept. The remaining dentin thickness was less than 0.5 mm and the specimens were stored wet. When one step self-etching adhesive is used, the semi-permeable adhesive layer could lead to water blistering at the interface.^61^ Slow self-curing under wet conditions might play a role in the poor adaptations in SC groups.

Under the conditions of this experiment, the exposure reciprocity rule was valid within a certain window of radiant exposure. The valid window of RU2 was 1.1–6.4 J/cm^2^ radiant exposure and that of RXU was 2–6.4 J/cm^2^ when PSSs were compared (Table 3). The validity of exposure reciprocity has been evaluated in some studies by various factors: the type and amount of photo initiator, resin viscosity including filler percentage, and matrix composition such as the bis-GMA:TEG-DMA ratio.^36,46,50^ In our experiment, different PSSs were found in the lowest and highest radiant exposure groups, which made the exposure reciprocity rule partially invalid (Table 3, Fig 4).

Longer light exposure can increase the radiant exposure and consequently lead to higher PSS. However, a prolonged light curing time under 3-mm-thick zirconia was not able to sufficiently polymerize the cement. Although the same radiant exposure was applied, the PSS-T3D99 could not reach the PSS-T0.5D20, even for a 99-s duration (Table 3, Fig 4). This result implies that low irradiance can lead to lower polymerization of resin cement, even though radiant exposure is augmented by prolonged exposure duration. One study showed that the degree of polymerization increases with longer exposure, but there is a theoretical radiant-exposure limit beyond which the degree of polymerization remains unchanged.^12^ If light of extremely low irradiance is applied, the opposite result is found. The PSS-T5D20 was lower than PSS-T3D7, despite the fact that the same radiant exposure was applied (Table 3, Fig 4). The explanation for this may be that the photo-initiators could not be activated by light of insufficient irradiance under 5-mm-thick zirconia. Some studies were conducted under very low radiant exposure or irradiance, and similar results were found.^18,25,46^ Previous studies state that for the exposure reciprocity law, there exists a minimum level of radiant exposure and exposure duration, which are material-specific.^12,46,51,54^

One of the limitations of using the SS-OCT device is the restoration design. Zirconia is usually used for tooth-coverage restoration; however, the scan dimension of the SS-OCT device was 5 x 5 mm. Therefore, the preparation was done for an inlay restoration of 3-mm diameter with diverse depths. The restoration was designed to be a simple cylinder for standardized comparison of interfacial adaptation. Another limitation may be that SS-OCT has a light-penetrating limit when capturing an image, which means that it cannot be used in deep cavities. The imaging depth of SS-OCT systems is known to be in the range of 2 mm.^56^ To overcome the limitations of observing thick specimens, the remaining dentin thickness under the cavity base was reduced to less than 0.5 mm, and the image was taken upside down to evaluate the restoration–tooth interface.^26^ The other limitation of using the SS-OCT device is its capability for detecting porosity in the cement space. A poorly polymerized resin cement would show voids with water inside. The SS-OCT can detect not only micro-gaps as a result of debonding but also bubbles or voids caused by poor polymerization or nonhomogeneous cement mixing.

The light distribution of the light-curing unit may affect the results of resin polymerization. Depending on the LCU, light emission can show a homogeneous or inhomogeneous distribution.^58^ If using an LCU with inhomogeneous light emission, polymerization of resin can vary by position or direction of the LCU tip. When resin composite is light cured with an LCU that delivers an inhomogeneous light emission, wear resistance and microhardness can be adversely affected.^23,58^ In terms of the wavelength of LCUs, the polymerization results using the single-wavelength LCU in this study could have been different had a multiple-wavelength LCU been used. Some LCUs have multiple-wavelength light-emitting diodes (LEDs) and others have single-wavelength LEDs. Studies have shown that multiple-wavelength LCUs produced inhomogeneous surface microhardness of resin composites, which depended on the short wavelengths of the LED (violet).^34,49^ Light transmittance through a restoration can show a greater decrease at short wavelengths, according to Rayleigh scattering,^30^ which could decrease the effectivity of violet light of an LCU.

Resin cement beneath a restoration is subject to different conditions than that of a direct resin composite. Some studies using very high irradiance (around 3000 mW/cm^2^) on direct resin composite showed the invalidity of exposure reciprocity, which may be because rapidly generated free radicals are prematurely spent by bimolecular termination.^17,18^ However, resin cement under a restoration is supposed to receive reduced light irradiance, which may not lead to sufficient polymerization of resin cement. For direct composite restorations, previous studies recommend LCUs of 1200–1500 mW/cm^2^ radiant exitance.^54,55^ Studies recommended 12–24 J/cm^2^ radiant exposure for direct composite resin of 2–4 mm thickness.^12,16,31,53^ Considering the reduced irradiance under restorations, irradiance and exposure duration should be carefully applied for zirconia cementation.

## Conclusion

Interfacial adaptation of translucent zirconia restoration differed according to restoration thickness, cement type, pretreatment method, and polymerization mode. The results of the pre-cure method showed similar or better interfacial adaptation than that of the co-cure method, depending on the thickness of the zirconia. The exposure reciprocity rule was valid within a certain window of radiant exposure. The radiant exposure window was different depending on the irradiance, exposure duration, and resin material.

## Clinical relevance:

When a thick zirconia restoration is luted with resin cement, light curing of adhesive is recommended before cement placement. Curing light intensity should be appropriately adapted for zirconia restorations, because low light intensity can lead to lower polymerization of resin cement.
